# The association between the Revised Cardiac Risk Index and short-term mortality after hip fracture surgery

**DOI:** 10.1007/s00068-020-01488-w

**Published:** 2020-09-17

**Authors:** Maximilian Peter Forssten, Ahmad Mohammad Ismail, Gabriel Sjolin, Rebecka Ahl, Per Wretenberg, Tomas Borg, Shahin Mohseni

**Affiliations:** 1 Department of Orthopedic Surgery, Orebro University Hospital, 701 85 Orebro, Sweden; 2 School of Medical Sciences, Orebro University, 702 81 Orebro, Sweden; 3 Division of Trauma and Emergency Surgery, Department of Surgery, Orebro University Hospital, 701 85 Orebro, Sweden; 4 Department of Surgery, Karolinska University Hospital, 171 76 Stockholm, Sweden; 5 Division of Surgery, CLINTEC, Karolinska Institutet, 171 76 Stockholm, Sweden; 6 Department of Surgery, Orebro University Hospital, 702 81 Orebro, Sweden

**Keywords:** Revised cardiac risk index, Hip fractures, Mortality, Risk assessment, Trauma

## Abstract

**Purpose:**

The post-operative mortality after hip fracture surgery is high and has remained largely unchanged during the last decades. The Revised Cardiac Risk Index (RCRI) is a tool used to evaluate the 30-day risk of, among other outcomes, post-operative mortality. The aim of this study is to determine the association between the RCRI score and post-operative mortality in patients undergoing hip fracture surgery.

**Methods:**

Data was obtained from the national hip fracture register which was cross-referenced with patients’ electronic hospital records. All adults who underwent primary emergency hip fracture surgery in Orebro County, Sweden, between January 1, 2013 and December 31, 2017, were included. Patients were divided into two cohorts: low RCRI (score = 0–1) and high RCRI (score ≥ 2). A Poisson regression model was employed to investigate the association between a high RCRI score and 30- and 90-day post-operative mortality.

**Results:**

A total of 2443 patients, of whom 446 (18%) had a high RCRI score, were included in the current study. When adjusting for age, sex, comorbidities and type of surgery, the incidence of 30-day mortality increased by 46% in the high RCRI cohort (adj. IRR 1.46, 95% CI, 1.10–1.94, *p* = 0.010). Similar results were observed for 90-day mortality (adj. IRR 1.50, 95% CI, 1.21–1.84, *p* < 0.001).

**Conclusion:**

The RCRI is applicable to patients that undergo surgery for traumatic hip fractures. A high RCRI score is associated with an increased incidence of both 30- and 90-day post-operative mortality. Future studies to evaluate these findings are needed.

## Introduction

Hip fractures are a common injury in the elderly. As the global population continues to age, the incidence of hip fractures is predicted to increase [1]. The risk of mortality after surgery for traumatic hip fractures is high, with rates around 10% and 16% for 30 and 90 days, respectively [2]. Despite several strategies employed during the last decades to alter the incidence of adverse outcomes, including new orthopedic innovations, fast-track programs and multidisciplinary teams, the post-operative mortality rate has remained largely unchanged [2, 3]. At the moment, there are few tools that are easy to use in clinical practice when trying to predict mortality rates after hip fracture surgery. Previous studies have suggested using tools such as the Possum Score, the Charlson Comorbidity Index, or the 5-factor modified frailty index [4–6]. However, these indices are complex or have inherent limitations which restrict their clinical utility; the Possum score has 18 variables, some of which are collected in the intra- or post-operative period [7], the Charlson Comorbidity Index has 17 variables [8], and the 5-factor modified frailty index includes functional status which is subjective and may be difficult to assess in the emergency setting [6]. The RCRI benefits from only requiring a few variables which can more easily be assessed on arrival to the emergency room. Although clinical decisions are made based on individual cases by the treating physician, predictive tools offer the benefit of facilitating resource allocation on a group level and they support physicians in objective decision-making as well as in communication with patients and family members.

The Revised Cardiac Risk Index (RCRI) is a tool used to evaluate the 30-day risk of post-operative myocardial infarction, cardiac arrest, and mortality [9]. The index is calculated using several pre-operative risk factors with an increasing score being associated with an increased risk of post-operative mortality [9–11]. RCRI has been extensively investigated and validated in general surgery and vascular surgery populations for its association with predicting mortality risks. However, many of the previous studies validating the RCRI have had a sample population predominantly composed of elective general surgical patients [12]. Its role in the emergency setting has been less thoroughly studied, particularly in hip fracture patients. It is important to emphasize the distinction between elective versus emergency surgical conditions, in the latter pre-operative optimization can be jeopardized by the time restriction for performing definitive treatment, i.e. surgery. The aim of this study is to determine the association between the RCRI score and 30-day mortality in patients undergoing emergency hip fracture surgery. The hypothesis is that a high RCRI score is associated with an increased incidence of 30-day mortality following hip fracture surgery.

## Materials and methods

The cohort was retrieved from the prospectively collected national quality register for hip fractures in Sweden, Rikshöft [13]. During the 5-year period between January 1, 2013 and December 31, 2017, all consecutive adult patients who underwent primary emergency hip fracture surgery in Orebro County, Sweden, were included in the current study. Orebro county consists of three hospitals: Orebro University Hospital and two university-affiliated regional hospitals. To keep the sample population homogenous, pathological hip fractures were excluded along with conservatively managed hip fractures. Pathological hip fractures have previously been associated with significantly increased rates of postoperative complications, readmissions, and deaths when compared to native hip fractures. Consequently, guidelines for native hip fractures might not be generalizable to pathological hip fracture patients [14]. Variables were obtained from both the national hip fracture register and patients’ electronic hospital records. The variables retrieved from the register were age, sex, date of hospital admission, American Society of Anesthesiologists (ASA) classification, type of fracture, surgical method, and date of hospital discharge. Comorbidity data was used to calculate both the Charlson Comorbidity Index (CCI) [8] and RCRI [15] for each patient. The CCI is based on the diagnosis of myocardial infarction, heart failure, peripheral vascular disease, cerebrovascular events, dementia, chronic obstructive pulmonary disease, connective tissue disease, peptic ulcer disease, liver disease, diabetes mellitus, hemiplegia, chronic kidney disease, cancer, leukemia, lymphoma, and AIDS. The patients’ electronic hospital records were reviewed in order to retrieve comorbidity data and time of death, as well as to supplement any data missing from the national hip fracture register.

### Calculating the Revised Cardiac Risk Index

The RCRI score is calculated based on the following variables: history of ischemic heart disease, history of congestive heart failure, history of cerebrovascular disease, pre-operative insulin therapy, pre-operative creatinine above 2 mg/dL and high-risk surgery, with each variable counting as 1 point if present. Hip fracture surgery is considered intermediate risk surgery according to the American College of Cardiology and the American Heart Association guidelines [16]; accordingly, points for high-risk surgery were not awarded to any patient in this study. Patients that had end-organ damage resulting from diabetes mellitus but lacked pre-operative insulin therapy were also awarded one point to reflect the severity of their diabetes.

### Statistical analysis

Patients were divided into two cohorts: low RCRI (score = 0–1) and high RCRI (score ≥ 2). Patient demographics and clinical characteristics were compared between the two cohorts. Categorical variables are reported with percentages while continuous variables are reported as mean and standard deviation (SD) or median and interquartile range (IQR). Pearson’s *χ*^2^ test and Fisher’s exact test were used to determine the statistical significance of differences between categorical variables. For continuous variables the Student’s *t*-test was used for normally distributed data, otherwise the Mann–Whitney *U* test was employed. The primary outcome of interest was 30-day post-operative mortality. The secondary outcome of interest was 90-day post-operative mortality. A Poisson regression model was employed to investigate the association between a high RCRI score and post-operative mortality. The analysis was performed while adjusting for age, sex, type of surgery and comorbidities that were not already included as part of the RCRI calculation. Results are reported as incidence rate ratios (IRR) for 30- and 90-day mortality with 95% confidence intervals (CI). Statistical significance was defined as a *p *value less than 0.05.

## Results

A total of 2443 patients met inclusion criteria. 48 patients had end-organ damage due to diabetes mellitus without ongoing insulin therapy; these patients were awarded one point on the RCRI resulting in 448 (18.3%) patients being included the high RCRI cohort. Females accounted for the majority of cases in both cohorts. Males were more prevalent in the high RCRI cohort compared to the low RCRI cohort (46.2% vs 29.7%, *p* < 0.001). The high RCRI cohort was older (mean age: 83.7 (SD 8.4) vs 81.5 (SD 10.2) years, *p* < 0.001), had more comorbidities (CCI ≥ 7: 77.5% vs. 19.6%, *p* < 0.001), and was less fit for surgery based on their American Society of Anesthesiologists classification (ASA ≥ 3: 82.0% vs. 44.1%, *p* < 0.001) (Table 1). For the majority of the comorbidities analyzed, a higher prevalence was seen in the high RCRI cohort as depicted in Table 2. No statistically significant difference was observed in the distribution of fracture types between the cohorts. The low RCRI cohort was managed with more advanced surgical techniques, such as total hip replacement and hemiarthroplasty, while the high RCRI cohort was subjected to less invasive surgical methods (Table 1).

**Table 1:** Demographics and clinical characteristics in patients before hip fracture surgery based on Revised Cardiac Risk Index (RCRI) score

	RCRI low^a^	RCRI high^b^	*p *value
	*N* = 1995	*N* = 448	
Age in years, mean [SD]	81.5 [10.2]	83.7 [8.4]	< 0.001
Sex, *n* (%)			< 0.001
Female	1403 (70.3)	241 (53.8)	
Male	592 (29.7)	207 (46.2)	
ASA classification, *n* (%)			< 0.001
1	180 (9.0)	4 (0.9)	
2	924 (46.3)	77 (17.2)	
3	785 (39.3)	278 (62.1)	
4	96 (4.8)	89 (19.9)	
Missing	10 (0.5)	0 (0.0)	
Charlson Comorbidity Index, *n* (%)			< 0.001
≤ 4	777 (38.9)	6 (1.3)	
5–6	827 (41.5)	95 (21.2)	
≥ 7	391 (19.6)	347 (77.5)	
Fracture type, *n* (%)			0.170
Non displaced cervical (Garden 1–2)	274 (13.7%)	47 (10.5)	
Displaced cervical (Garden 3–4)	720 (36.1)	152 (33.9)	
Basicervical	87 (4.4)	24 (5.4)	
Peritrochanteric (two fragments)	475 (23.8)	112 (25.0)	
Peritrochanteric (multiple fragments)	296 (14.8)	83 (18.5)	
Subtrochanteric	143 (7.2)	30 (6.7)	
Type of surgery, *n* (%)			0.003
Screws or pins	398 (19.9)	93 (20.8)	
Screws or pins with sideplate	703 (35.2)	179 (40.0)	
Intramedullary nail	319 (16.0)	82 (18.3)	
Hemiarthroplasty	413 (20.7)	78 (17.4)	
Total hip replacement	162 (8.1)	16 (3.6)	

*ASA* American Society of Anesthesiologists, *N* total number of patients

^a^RCRI score 0–1

^b^RCRI score ≥ 2

**Table 2:** Preoperative co-morbidities in patients before hip fracture surgery based on Revised Cardiac Risk Index (RCRI) score

	RCRI low^a^	RCRI high^b^	*p *value
	*N* = 1995	*N* = 448	
Myocardial infarction, *n* (%)	118 (5.9)	262 (58.5)	< 0.001
Heart failure, *n* (%)	144 (7.2)	282 (62.9)	< 0.001
Peripheral vascular disease, *n* (%)	104 (5.2)	76 (17.0)	< 0.001
Cerebrovascular event, *n* (%)	322 (16.1)	273 (60.9)	< 0.001
Dementia, *n* (%)	478 (24.0)	96 (21.4)	0.280
Chronic obstructive pulmonary disease, *n* (%)	157 (7.9)	66 (14.7)	< 0.001
Connective tissue disease, *n* (%)	29 (1.5)	11 (2.5)	0.190
Peptic ulcer disease, *n* (%)	184 (9.2)	65 (14.5)	< 0.001
Liver disease, *n* (%)	26 (1.3)	5 (1.1)	0.930
Diabetes Mellitus, *n* (%)			< 0.001
Uncomplicated	113 (5.7)	48 (10.7)	
End-organ damage	92 (4.6)	119 (26.6)	
Hemiplegia, *n* (%)	36 (1.8)	33 (7.4)	< 0.001
Chronic kidney disease, *n* (%)	80 (4.0)	97 (21.7)	< 0.001
Cancer, *n* (%)			0.046
Local tumor	379 (19.0)	107 (23.9)	
Metastatic	56 (2.8)	15 (3.3)	
Leukemia, *n* (%)	11 (0.6)	6 (1.3)	0.130
Lymphoma, *n* (%)	13 (0.7)	0 (0.0)	0.180

*N* total number of patients

^a^RCRI score 0–1

^b^RCRI score ≥ 2

The incidence of 30-day mortality was higher in the high RCRI cohort (13.4% vs 7.1%, *p* < 0.001). The same was observed for unadjusted 90-day mortality (22.5% vs 12.7%, *p* < 0.001) (Table 3). An increase in the incidence of mortality with increasing RCRI scores was observed for both 30- and 90-day mortality (Table 4). Multivariable Poisson regression analysis found that increasing age, male sex, dementia, liver disease and metastatic cancer were associated with significantly increased incidences of both 30- and 90-day post-operative mortality after hip fracture surgery. When adjusting for age, sex, comorbidities and type of surgery, the incidence of 30-day mortality increased by 46% in the high RCRI cohort (adj. IRR 1.46, 95% CI, 1.10–1.94, *p* = 0.010). Similar results were observed for 90-day mortality (adj. IRR 1.50, 95% CI, 1.21–1.84, *p* < 0.001) (Table 5).

**Table 3:** Outcomes in patients with hip fractures based on Revised Cardiac Risk Index (RCRI) score

	RCRI low^a^	RCRI high^b^	*p *value
	*N* = 1995	*N* = 448	
Hospital length of stay in days, median [Q2, Q3]	8.0 [5.0, 11.0]	9.0 [5.8, 13.0]	< 0.001
30-day mortality, *n* (%)	141 (7.1)	60 (13.4)	< 0.001
90-day mortality, *n* (%)	253 (12.7)	101 (22.5)	< 0.001

*N* total number of patients

^a^RCRI score 0–1

^b^RCRI score ≥ 2

**Table 4:** The distribution of Revised Cardiac Risk Index (RCRI) score in patients with hip fractures for 30- and 90-day post-operative mortality

	Total	30-day mortality	90-day mortality
	*N* = 2443	*N* = 201	*N* = 354
RCRI 0, *n* (%)	1235 (50.6)	75 (6.1)	133 (10.8)
RCRI 1, *n* (%)	760 (31.1)	66 (8.7)	120 (15.8)
RCRI 2, *n* (%)	319 (13.1)	45 (14.1)	72 (22.6)
RCRI 3, *n* (%)	108 (4.4)	13 (12.0)	24 (22.2)
RCRI 4, *n* (%)	20 (0.8)	2 (10.0)	5 (25.0)
RCRI 5, *n* (%)	1 (0.0)	0 (0.0)	0 (0.0)

*N* total number of patients

**Table 5:** Incidence rate ratio (IRR) for 30- and 90-day mortality after hip fracture surgery

Variable	30-day IRR (95% CI)	*p *value	90-day IRR (95% CI)	*p* value
Revised Cardiac Risk Index				
Low^a^	ref		ref	
High^b^	1.46 (1.10–1.94)	0.010	1.50 (1.21–1.84)	< 0.001
Age	1.07 (1.05–1.09)	< 0.001	1.07 (1.05–1.08)	< 0.001
Sex				
Female	ref		ref	
Male	2.38 (1.81–3.14)	< 0.001	1.67 (1.36–2.03)	< 0.001
Peripheral vascular disease				
No	ref		ref	
Yes	0.83 (0.49–1.40)	0.485	0.96 (0.69–1.33)	0.798
Dementia				
No	ref		ref	
Yes	2.26 (1.73–2.94)	< 0.001	2.26 (1.87–2.73)	< 0.001
Chronic obstructive pulmonary disease				
No	ref		ref	
Yes	1.35 (0.89–2.06)	0.162	1.28 (0.94–1.76)	0.123
Connective tissue disease				
No	ref		ref	
Yes	1.22 (0.58–2.57)	0.595	0.72 (0.33–1.58)	0.413
Liver disease				
No	ref		ref	
Yes	2.63 (1.02–6.73)	0.045	2.28 (1.11–4.67)	0.025
Cancer				
None	ref		ref	
Local tumor	1.27 (0.95–1.70)	0.101	1.19 (0.95–1.48)	0.125
Metastatic	2.46 (1.46–4.13)	0.001	3.07 (2.15–4.40)	< 0.001
Leukemia				
No	ref		ref	
Yes	2.18 (0.63–7.52)	0.218	1.31 (0.40–4.30)	0.653
Lymphoma				
No	ref		ref	
Yes	N/A^c^	–	0.62 (0.11–3.46)	0.583
Type of surgery				
Screws or pins	ref		ref	
Screws or pins with sideplate	1.12 (0.79–1.59)	0.530	1.22 (0.95–1.57)	0.117
Intramedullary nail	1.17 (0.77–1.76)	0.462	1.05 (0.77–1.43)	0.761
Hemiarthroplasty	0.90 (0.60–1.36)	0.608	0.96 (0.71–1.30)	0.791
Total hip replacement	0.64 (0.23–1.82)	0.405	0.57 (0.25–1.29)	0.174

Poisson regression model with robust standard errors. Model adjusted for age, sex, comorbidities and type of surgery

^a^Revised Cardiac Risk Index score 0–1

^b^Revised Cardiac Risk Index score ≥ 2

^c^No deaths were registered in patients with lymphoma within 30 days post-operatively

## Discussion

This is the first large-scale study investigating the association between the Revised Cardiac Risk Index score and short-term mortality after hip fracture surgery. A significant increase in the incidence of both 30- and 90-day mortality was detected in patients with a high RCRI score. This relationship remained significant after adjusting for age, sex, type of surgery and relevant comorbidities, with an almost 50% increase in the incidence of mortality in patients with a high RCRI score compared to a low RCRI score.

Hip fractures predominantly occur in the elderly who tend to be burdened by several comorbidities [2, 17–20]. This is supported by the demographics of the sample population in the current study. Despite advances in the care of hip fracture patients, mortality rates have remained high during the last decades [2, 3]. Gundel et al. [2] published a study in 2019, composed of 113,721 patients, which calculated the 30- and 90-day post-operative mortality rates after hip fracture surgery to be 9.6% and 16%, respectively. These results mirror the current study where the overall 30- and 90-day post-operative mortality rates were 8.2% and 14.5%, respectively. Gundel et al. [2] also demonstrated that male sex, increasing age and comorbidities were significant risk factors for 90-day post-operative mortality in hip fracture patients, an association that is in line with earlier studies [2, 17–20]. This remained true for our cohorts where these same variables were associated with an increased incidence of post-operative mortality.

The RCRI was developed by Lee et al. [15] and is now widely used to estimate the 30-day risk of myocardial infarction, cardiac arrest and mortality after non-cardiac surgery [9]. Several studies have investigated the relationship between the RCRI and mortality [9, 10]. Lindenauer et al. specifically outlined a proportional relationship between in-hospital mortality rate and the RCRI score; with an increased mortality at higher RCRI scores [11]. A meta-analysis published in 2010 by Ford et al. validated the previous conclusion that the RCRI could predict adverse cardiac events after non-cardiac surgery. Nevertheless, they did not distinguish between different types of non-cardiac surgery with the exception of one subgroup analysis for vascular surgery specifically, where the RCRI exhibited a low predictive value [12]. However, subsequent studies have provided additional evidence for the RCRI’s ability to predict adverse events in patients undergoing non-cardiac surgery [21, 22].

There is only one previous study composed of 422 patients, conducted by Guerra et al. [23] investigating the association between RCRI and mortality in hip fracture patients. Their study could not demonstrate a predictive relationship between the RCRI and 1-year mortality. The difference between their results and the results of the current study may be attributed to the limited size of their sample population and their use of 1-year rather than short-term mortality. Additionally, the current study divides patients into two cohorts based on RCRI scores as well as adjusts for relevant comorbidities and other clinically important factors that could affect the risk of mortality; this was not carried out by Guerra et al. [23].

In general, less invasive surgical techniques are preferred for frail, elderly patients [24–26]. This is reflected in our data in which hemiarthroplasties were 20% more common and total hip replacements were 125% more common in the low RCRI cohort, which were younger, less burdened by comorbidities, and more fit for surgery, based on their CCI and ASA scores respectively. More invasive techniques tend to result in more expansive tissue damage along with a greater inflammatory response which is postulated to increase the post-operative mortality risk due to systemic inflammatory conditions and subsequent multi-organ failure, most notably from cardiac and respiratory origin [27–29]. Nevertheless, none of the surgical techniques were associated with an increase or decrease in the incidence of mortality in our study cohorts. This observation may be the result of the appropriate clinical decision having been made by the attending orthopedic surgeons, where more frail patients were allocated to less invasive and stressful surgery.

The ability to evaluate hip fracture patients pre-operatively may be of value when trying to achieve a reduction in post-operative mortality. Since a high RCRI score appears to be associated with an increased incidence of both 30- and 90-day mortality after hip fracture surgery, its application may be considered in order to achieve better resource allocation, patient prioritization, surgical method selection and detection of patients in need of extended pre-operative optimization. More importantly, it may help orthopedic surgeons and their multidisciplinary teams to identify those patients in need of higher post-operative care levels in order to prevent adverse post-operative cardiac events. Consequently, the RCRI could be considered for inclusion in surgical care protocols for patients undergoing emergency orthopedic surgery. The authors hope that the results of the current study will encourage further research in this field of orthopedic trauma surgery in order to determine the viability of utilizing the RCRI in this manner for patients suffering from hip fractures.

The current study has both strengths and limitations that need to be mentioned. To the authors' knowledge, this is the first study investigating the association between the RCRI and short-term mortality, based *exclusively* on patients subjected to emergency surgery for traumatic hip fractures. Furthermore, the results are strengthened by the fact that patient management remained uniform, since all patients were treated within the same orthopedic department, and that the register data could be cross-referenced with the electronic hospital charts. The use of register data resulted in certain limitations as the authors were unable to determine the cause of death for any patients included in the study. Nevertheless, this is not a necessity since the RCRI includes mortality in its outcome prediction, which is not solely exclusive to cardiovascular events [9]. Analyses regarding prioritization for surgery and pre-operative optimization could not be performed since this information was not readily available and requires a prospective study design.

## Conclusion

The Revised Cardiac Risk Index (RCRI) is applicable to patients that undergo surgery for emergency traumatic hip fractures when assessing the incidence of short-term post-operative mortality. A high RCRI score is associated with an increased incidence of both 30- and 90-day post-operative mortality. Further prospective studies are nevertheless required to evaluate the utility of the RCRI as a predictor of mortality in hip fracture patients.

## Data Availability

All codes are available for retrieval upon reasonable request.

## Availability of data and material

All data is available for retrieval upon reasonable request.

## References

[1] Kanis JA, Oden A, McCloskey EV, Johansson H, Wahl DA, Cooper C (2012). A systematic review of hip fracture incidence and probability of fracture worldwide. Osteoporos Int.

[2] Gundel O, Thygesen LC, Gogenur I, Ekeloef S (2019). Postoperative mortality after a hip fracture over a 15-year period in Denmark: a national register study. Acta Orthop..

[3] Kates SL (2016). Hip fracture programs: are they effective?. Injury.

[4] Bonicoli E, Parchi P, Piolanti N, Andreani L, Niccolai F, Lisanti M (2014). Comparison of the POSSUM score and P-POSSUM score in patients with femoral neck fracture. Musculoskelet Surg.

[5] Flikweert ER, Wendt KW, Diercks RL, Izaks GJ, Landsheer D, Stevens M (2018). Complications after hip fracture surgery: are they preventable?. Eur J Trauma Emerg Surg.

[6] Traven SA, Reeves RA, Althoff AD, Slone HS, Walton ZJ (2019). New five-factor modified frailty index predicts morbidity and mortality in geriatric hip fractures. J Orthop Trauma.

[7] Copeland GP, Jones D, Walters M (1991). POSSUM: a scoring system for surgical audit. Br J Surg.

[8] Charlson ME, Pompei P, Ales KL, MacKenzie CR (1987). A new method of classifying prognostic comorbidity in longitudinal studies: development and validation. J Chronic Dis.

[9] Duceppe E, Parlow J, MacDonald P, Lyons K, McMullen M, Srinathan S (2017). Canadian Cardiovascular Society guidelines on perioperative cardiac risk assessment and management for patients who undergo noncardiac surgery. Can J Cardiol.

[10] Devereaux PJ, Goldman L, Cook DJ, Gilbert K, Leslie K, Guyatt GH (2005). Perioperative cardiac events in patients undergoing noncardiac surgery: a review of the magnitude of the problem, the pathophysiology of the events and methods to estimate and communicate risk. CMAJ.

[11] Lindenauer PK, Pekow P, Wang K, Mamidi DK, Gutierrez B, Benjamin EM (2005). Perioperative beta-blocker therapy and mortality after major noncardiac surgery. N Engl J Med.

[12] Ford MK, Beattie WS, Wijeysundera DN (2010). Systematic review: prediction of perioperative cardiac complications and mortality by the revised cardiac risk index. Ann Intern Med.

[13] Rikshoft. Swedish National Registry of hip fracture patient care. https://rikshoft.se/about-rikshoft/. Accessed January 29 2020.

[14] Amen TB, Varady NH, Hayden BL, Chen AF (2020). Pathologic versus native hip fractures: comparing 30-day mortality and short-term complication profiles. J Arthroplasty.

[15] Lee TH, Marcantonio ER, Mangione CM, Thomas EJ, Polanczyk CA, Cook EF (1999). Derivation and prospective validation of a simple index for prediction of cardiac risk of major noncardiac surgery. Circulation.

[16] Fleisher LA, Beckman JA, Brown KA, Calkins H, Chaikof E, Fleischmann KE (2007). ACC/AHA 2007 guidelines on perioperative cardiovascular evaluation and care for noncardiac surgery: a report of the American College of Cardiology/American Heart Association Task Force on Practice Guidelines (Writing Committee to Revise the 2002 Guidelines on Perioperative Cardiovascular Evaluation for Noncardiac Surgery): developed in collaboration with the American Society of Echocardiography, American Society of Nuclear Cardiology, Heart Rhythm Society, Society of Cardiovascular Anesthesiologists, Society for Cardiovascular Angiography and Interventions, Society for Vascular Medicine and Biology, and Society for Vascular Surgery. Circulation.

[17] Cher EWL, Allen JC, Howe TS, Koh JSB (2019). Comorbidity as the dominant predictor of mortality after hip fracture surgeries. Osteoporos Int.

[18] Choi HG, Lee YB, Rhyu SH, Kwon BC, Lee JK (2018). Mortality and cause of death postoperatively in patients with a hip fracture: a national cohort longitudinal follow-up study. Bone Joint J..

[19] Panula J, Pihlajamaki H, Mattila VM, Jaatinen P, Vahlberg T, Aarnio P (2011). Mortality and cause of death in hip fracture patients aged 65 or older: a population-based study. BMC Musculoskelet Disord.

[20] Schnell S, Friedman SM, Mendelson DA, Bingham KW, Kates SL (2010). The 1-year mortality of patients treated in a hip fracture program for elders. Geriatr Orthop Surg Rehabil.

[21] Andersson C, Wissenberg M, Jorgensen ME, Hlatky MA, Merie C, Jensen PF (2015). Age-specific performance of the revised cardiac risk index for predicting cardiovascular risk in elective noncardiac surgery. Circ Cardiovasc Qual Outcomes.

[22] Hwang JW, Kim EK, Yang JH, Chang SA, Song YB, Hahn JY (2015). Assessment of perioperative cardiac risk of patients undergoing noncardiac surgery using coronary computed tomographic angiography. Circ Cardiovasc Imaging..

[23] Guerra MTE, Giglio L, Morais JMM, Labatut G, Feijo MC, Kayser CEP (2019). The Relationship between the Lee Score and Postoperative Mortality in Patients with Proximal Femur Fractures. Rev Bras Ortop (Sao Paulo).

[24] Ding T, Zhang B, Tian S, Wang Y, Sun K (2018). Selection principles and application status of surgical methods for hip fracture in the elderly. Zhongguo Xiu Fu Chong Jian Wai Ke Za Zhi.

[25] Nicolaides V, Galanakos S, Mavrogenis AF, Sakellariou VI, Papakostas I, Nikolopoulos CE (2011). Arthroplasty versus internal fixation for femoral neck fractures in the elderly. Strategies Trauma Limb Reconstr.

[26] Tidermark J, Ponzer S, Svensson O, Soderqvist A, Tornkvist H (2003). Internal fixation compared with total hip replacement for displaced femoral neck fractures in the elderly. A randomised, controlled trial. J Bone Joint Surg Br..

[27] Desborough JP (2000). The stress response to trauma and surgery. Br J Anaesth.

[28] Loftus TJ, Efron PA, Moldawer LL, Mohr AM (2016). beta-Blockade use for traumatic injuries and immunomodulation: a review of proposed mechanisms and clinical evidence. Shock.

[29] Moor D, Aggarwal G, Quiney N (2017). Systemic response to surgery. Surgery (Oxford).

